# Age-Based Hiring Discrimination as a Function of Equity Norms and Self-Perceived Objectivity

**DOI:** 10.1371/journal.pone.0084752

**Published:** 2014-01-21

**Authors:** Nicole M. Lindner, Alexander Graser, Brian A. Nosek

**Affiliations:** 1 DataSong, Irvine, California, United States of America; 2 University of Regensburg, Regensburg, Germany; 3 Departement of Psychology, University of Virginia, Charlottesville, Virginia, United States of America; 4 Center for Open Science, Charlottesville, Virginia, United States of America; University of Minnesota, United States of America

## Abstract

Participants completed a questionnaire priming them to perceive themselves as either objective or biased, either before or after evaluating a young or old job applicant for a position linked to youthful stereotypes. Participants agreed that they were objective and tended to disagree that they were biased. Extending past research, both the objective and bias priming conditions led to an *increase* in age discrimination compared to the control condition. We also investigated whether equity norms reduced age discrimination, by manipulating the presence or absence of an equity statement reminding decision-makers of the legal prohibitions against discrimination “on the basis of age, disability, national or ethnic origin, race, religion, or sex.” The presence of equity norms increased enthusiasm for both young and old applicants when participants were not already primed to think of themselves as objective, but did not reduce age-based hiring discrimination. Equity norms had no effect when individuals thought of themselves as objective – they preferred the younger more than the older job applicant. However, the presence of equity norms did affect individuals’ perceptions of which factors were important to their hiring decisions, increasing the perceived importance of applicants’ expertise and decreasing the perceived importance of the applicants’ age. The results suggest that interventions that rely exclusively on decision-makers' intentions to behave equitably may be ineffective.

## Introduction

Age-Based Hiring Discrimination as a Function of Equity Norms and Self-Perceived Objectivity. Older adults experience discrimination in a variety of contexts, including employment [1]. In the United States, the federal Age Discrimination in Employment Act (ADEA) prohibits discrimination against older workers in important contexts, including hiring, termination, and financial compensation [2]. Although this legislation protects currently-employed older adults from being fired because of their age, it appears to discourage employers from *hiring* older adults in the first place. Lahey [2] compared variations across the United States in employment outcomes for older male workers since the ADEA has been enforced; older men were less likely to be hired in states where it was easier to file age discrimination lawsuits. Furthermore, employers for entry-level jobs were over 40% less likely to interview equally-qualified older applicants than young applicants, despite resumes with stereotype-disconfirming details [3]. Age discrimination appears be influenced by negative stereotypes of older adults’ such as lack of adaptability [4], lack of competence, less attractive, and more old-fashioned [5], [6], [7]. However, age discrimination in hiring occurs despite a lack of age-related declines in work performance [8], [9] and the economic benefits of a workplace with older, more experienced workers [10]. The present research contrasted two interventions that are intended to reduce hiring discrimination by altering decision-makers’ sense of their own objectivity or the environmental norms prohibiting discrimination.

### Perceptions of the Self as Objective or Biased

One intuitive way to reduce discrimination could be to remind people that they care about and consider themselves to be objective. However, previous research finds that individuals primed with a sense of *personal objectivity* are more likely to act on stereotypical beliefs, resulting in discrimination against women in a hiring context [11]. This apparently ironic effect occurs because priming an identity as objective experientially suggests that one’s thoughts must come from an objective source. Thus, individuals are more willing to act on whatever thoughts come to mind, including their stereotypical thoughts. As evidence for this interpretation, a sense of personal objectivity increased gender discrimination among participants who were high rather than low in gender stereotyping and among participants who had been subtly primed with gender stereotypes [11]. We investigated whether the opposite would also hold – whether individuals who were primed to think of themselves as being imperfectly objective (i.e., biased) would be *less* likely to express hiring discrimination against older adults. Because individuals view *their own* judgments as much less subject to biases than *others*’ judgments (known as the bias blind spot) [12], [13], we intended to induce individuals to consider that their judgments could occasionally be biased.

### Presence of Equity Norms, as Indicated by Nondiscrimination Statutes

Another common approach to increasing interest in objectivity and non-discrimination is to remind people of the legal or normative standards. As such, we also manipulated the presence or absence of *equity norms*. It is common practice to establish equity norms for hiring new employees by stating nondiscrimination statutes. Despite their prevalence, experimental research has focused on their effects on job applicants [14], [15], including older applicants [16], rather than on employment decision-makers. We hypothesized that the presence of equity norms might not achieve their intended purpose to reduce discrimination. This is suggested by theory [17] that posits that correcting for unwanted biases requires that all of the following sequence of events be achieved: individuals must be aware that the bias could influence their decision, be motivated to correct the bias, know the degree to which the bias influences their decision, and have the mental control necessary to adjust their decision. That is, being reminded of nondiscrimination statutes could increase individuals' motivation to evaluate equitably without influencing their judgments, to the extent that individuals are (a) either unaware of how their preexisting social-group biases influence their judgments or (b) are unable to correct their judgments.

Because most people already believe that they are objective [11], [18], we hypothesized that the presence of equity norms would not reduce the age difference in hiring evaluations. Based on prior evidence, we hypothesized that the presence of equity norms might instead change what information people *believed* that they used in their hiring decisions, even when this was not actually the case. Research has found that individuals “construct” hiring and other selection criteria to justify their decisions as fair [19], [20]. For example, decision-makers who hired male rather than female applicants justified this decision by inflating the importance of whichever qualifications favored the male applicant [19].

## Materials and Methods

### Ethics Statement

The University of Virginia Institutional Review Board for the Social and Behavioral Sciences approved this research and informed consent process (#2003–0173). Participants provided written informed consent prior to participation, and received a written debriefing at the end of the study session.

### Participants

Adult participants were randomly assigned to this experiment from a pool of available studies at Project Implicit (https://implicit.harvard.edu). These samples are not representative of the general population; selection biases influence whether individuals learn about the website, choose to visit it, register at the research-site, and complete the attitude measures. Even so, the sample’s size and demographic heterogeneity (especially for age, compared to psychology participant pools) permit greater confidence that these effects generalize to working adults than typical undergraduate samples. 1,852 individuals consented to participate, and 1,590 participants completed at least one of the outcome measures. Consenting participants (*M*_age_ = 30.2, *SD* = 12.7, range  = 18 – 80) were predominantly women (65%), US citizens or residents (83%), and educated (89% had some college education or more). Sample sizes vary for tests as a function of missing data.

Individuals who were assigned to the study and chose not to participate did not significantly differ in age (measured in years) from either consenters, *t*(2091)  = 0.35, *p* = .725, or participants who completed an outcome measure, *t*(1829)  = 0.65, *p* = .518, nor did these noncompleters significantly differ on political orientation or education level (all *t*s ≤ 1.65). The gender composition of non-responders (72% female) differed slightly from participants who completed an outcome measure (65% female), χ^2^(1)  = 4.11, *p* = .043.

## Materials

**Self-perceptions priming.** In the experimental conditions, we primed participants with a sense of personal objectivity or bias by asking them to respond to a self-perceptions questionnaire, using a 6-point agree-disagree scale (–2.5 =  *disagree strongly*, 2.5 =  *agree strongly*). As in previous research [11], participants in the *self-perceived objectivity* condition completed five randomly-ordered questionnaire items (“In most situations, I try to do what seems reasonable and logical”; “When forming an opinion, I try to objectively consider all of the facts”; “My judgments are based on a logical analysis of the facts”; “My decision making is rational and objective”; and “When making a decision, I even-handedly weigh all of the relevant evidence”). The *self-perceived bias* condition paralleled the objectivity condition. The questionnaire items were intended to elicit participants’ agreement that their decision making could occasionally be less than fully objective. It presented five items in randomized order (“Once in a while, I have trouble doing what might be reasonable and logical”; “When forming an opinion, I do not always objectively consider all of the facts”; “My judgments are occasionally influenced by my own preconceptions, rather than just the facts”; “My decision making can be influenced by my personal biases”; and “When making a decision, I occasionally just go with what I want, rather than even-handedly weighing all of the relevant evidence”).

**Equity norms manipulation.** We manipulated whether the instructions for the hiring decision concluded with the following sentence: “Keep in mind that hiring managers must follow state and federal laws prohibiting discrimination on the basis of age, disability, national or ethnic origin, race, religion, or sex.” This paralleled statements common to employment contexts that assert companies’ compliance with nondiscrimination statutes and embedded age within six social categories.

**Hiring scenario.** Discrimination is thought to be more likely when there is a mismatch between group stereotypes and the evaluation context (as has been shown for age discrimination) [4]. We adapted a paradigm from Uhlmann and Cohen [11] that examined hiring discrimination via gender stereotypes to be relevant to age stereotypes. Participants were presented with description of a trendy company, an available marketing manager position, and a summary of information from a job application (see Supporting Materials S1). The company description and position evoked youthful stereotypes (hip, innovative). The male applicant was described as either 31 or 54 years old, and had some experience in a comparable company. Also, he was described by recommenders with one of four descriptions (see Table 1), containing four positive (e.g., “Excellent capacity to quickly grasp new theories”) and three somewhat negative (e.g., “Disorganization sometimes hinders productivity”) phrases, varying the domain of the phrases across the four counterbalanced descriptions (i.e., dependability, creativity, interpersonal skills, leadership skills, or organization).

**Table 1:** Content of recommendation letter highlights for 4 counterbalanced job applicants.

C#	Highlights:	Highlights:
	Recommendation Letter 1	Recommendation Letter 2
#1	Was promoted to a position utilizing his interpersonal skills	• Interacts and gets along well with subordinates, superiors, and clients
	• Sometimes has difficulty making decisions	• Punctual and typically exceeds expectations
	• Voluntarily works overtime and takes work home to meet deadlines	• Occasionally hesitant in making a final decision regarding various subordinates’ ideas
	• Adequate performance in increasing departmental profits	
#2	• Has the analytical skills to identify needs and devise viable solutions	• Praised for how he handles pressure
	• Required occasional guidance and mentorship in maintaining good relations with subordinates	• Excellent capacity to quickly grasp new theories
	• Praised for being unflustered and productive during frenzied periods	• Still improving grasp of interpersonal skills required for management
	Acceptable leadership skills	
#3	• Somewhat conservative in promoting/approving edgy marketing appeals	• Extremely positive and dependable employee
	• Remains steadfast in his cheerfulness, calmness, and dependability	• Exceeds expectations in interpersonal skills and dependability, but less so in creativity and vision
	• Interacts and gets along well with fellow employees and superiors, as well as clients	• Attentive to tasks and works tirelessly to achieve the goals of the department
	• Adequate, but not exceptional productivity	
#4	• Always willing to offer assistance and has an excellent rapport with employees and clients	• Enjoys good relationships with employees and encourages their creativity
	• Occasionally late for work and meetings	• Productivity is occasionally hampered by lateness
	• Excellent capacity to quickly grasp new theories and creatively generate related ideas	• Praised for innovative ideas in previous projects
	• Disorganization sometimes hinders productivity	

*Note.* The job applicant was described with one of four profiles of recommender highlights. These profiles were randomly-assigned across participants. Hiring evaluations were altered as a main effect of which profile was used to describe the job applicant, *F*(3, 1584)  = 6.79, *p* = .0001, *R* ^2^ = .013. But critically, the profile type never significantly interacted with the manipulated factors, as self-perceptions manipulations, the presence of equity norms, and the applicants’ age (all *F*s ≤ 2.23). Nor did it interact significantly with the manipulated factors in predicting the perceived importance of the applicants’ age (all *F*s ≤ 1.49) or the perceived importance of the applicants’ expertise (all *F*s ≤ 1.85).

Three items assessed the extent to which participants believed that the applicant would be successful (1 =  *Not at all successful*, 6 =  *Extremely successful*), was a good fit (1 =  *Extremely bad fit*, 6  =  *Extremely good fit*), and should be hired (1  =  *Should definitely not be hired*, 6  =  *Should definitely be hired*).

**Importance of decision factors.** After the hiring decision, participants reported how important each of several factors had been in deciding whether to hire the applicant (1  =  *Not at all important*, 7  =  *Essential to my decision*). The factors were presented in randomized order and included both normative factors (*work experience*, *educational background*, *expertise*, *creativity*, *interpersonal skills*, *dependability and productivity*, and *leadership skills*) and demographic characteristics (*age*, *marital and parental status*).

### Design and Procedure

The experiment consisted of 3 (Self-perceived objectivity vs. Self-perceived bias vs. Control) × 2 (Equity statement vs. Control) × 2 (Young vs. Old job applicant) between-participants design; the four applicant descriptions were also randomized across participants, and did not interact with the experimental effects described below. Participants were randomly assigned either to the control condition or the experimental conditions of self-perceived objectivity or self-perceived bias. In the experimental conditions, participants began the experiment by responding to one of the self-perceptions questionnaires and then completed the hiring decision. In the control condition, participants began with the hiring decision and then completed one of the self-perceptions questionnaires. In the hiring decision, participants were presented with a company description, a job position, and applicant materials; the applicant was described either as young or old, and a sentence reminding participants of nondiscrimination statutes (equity norms) was either present or absent. Participants evaluated the applicant’s suitability for the position and then reported the importance of several factors to their decision. All materials and data are available for download at: https://osf.io/ie4md/.

### Analysis Strategy

A general linear model (GLM) framework was used to test the hypotheses. Participants who responded to at least one of the three hiring decision items were including in the analyses, including the small number of participants who failed to respond to all (*n* = 39) or some (*n* = 4) of the self-perceptions manipulation items (37 of these 43 participants were in the control condition, completing the self-perceptions manipulation after the hiring decision).

## Results

### Effectiveness of the Self-Perceptions Manipulations

The five items for each scale were averaged to form an index of individuals’ self-perceived objectivity (Cronbach’s α = .86) or their self-perceived bias (α = .76). Participants who were primed with the self-perceived objectivity items before making the hiring decision (*M* = 1.38) did not differ in self-perceived objectivity from participants who completed the items after the hiring decision (control participants; *M* = 1.39), *t*(781)  = 0.09, *p* = .928, Cohen’s *d* = 0.01. Participants who were primed with the self-perceived bias items *before* the hiring decision (*M* = 0.10) saw themselves as slightly more biased than participants who completed the items after the hiring decision (control participants; *M* = –0.11), *t*(764)  = 2.95, *p* = .003, *d* = 0.23. This suggests that participants engaged in post-decision defensiveness of their own objectivity.

Comparing agreement between the self-perceived objectivity and bias questionnaires indicates that on average, individuals agreed that they were objective (*M* = 1.38), but failed to agree that they were biased (*M* = 0.04), *t*(1547)  = 31.37, *p<*.001, *d* = 1.59. On average, 96% of participants agreed at least slightly that their decision-making was objective, while only 47% agreed at least slightly that their decision-making could be subject to bias. This large difference in agreement occurred even though the bias items were designed to be very mild. This undermines the goal of the “self-perceived bias” manipulation. Indeed, as reported next, the “objective” and “bias” manipulation conditions had similar effects on hiring discrimination, suggesting that both reinforced a sense of objectivity.

### Context Sensitivity of Hiring Discrimination

The three items used to evaluate applicant suitability for the position were averaged (α = .90). Participants slightly preferred the younger applicant, such that participants were more willing to hire the young job applicant (*M* = 4.14) than the older job applicant (*M* = 4.00), *t(*1586)  = 2.77, *p* = .006, *d* = 0.14. Overall, hiring decisions (*R* ^2^ = .011) were predicted by the main effect of applicants’ age, *F(*1, 1582)  = 7.72, *p* = .006, partial-η^2^ (η_p_^2^)  = .005, but not as a main effect of the self-perceptions priming, *F*(2, 1582)  = 0.46, *p* = .630, η_p_^2^ = .001. Importantly, the anticipated interaction between the self-perceptions priming and the applicants’ age emerged, *F*(2, 1582)  = 4.67, *p* = .001, η_p_^2^ = .006. This pattern persisted (*R* ^2^ = .049) when including participants’ own age as a predictor, *F*(1, 1581)  = 62.60, *p<*.001, η_p_^2^  = .038. Older adults were less willing to hire all job applicants, *B* = –.015, *SE* = .002, *t*(1581)  = 7.91, *p<*.001, regardless of the applicants’ age or the self-perceptions priming condition, such that participants’ age did not interact with any manipulated factors, *F*s ≤ 1.

Follow-up analysis of the simple main effect of the applicants’ age within each of the self-perceptions priming conditions indicated that in the self-perceived objectivity priming condition, participants favored the young applicant (*M* = 4.23) over the older applicant (*M* = 3.95), *t*(1582)  = 3.21, *p* = .001, *d* = 0.28, as did participants in the self-perceived bias priming condition, (*M*s = 4.18, 3.97, respectively), *t*(1582)  = 2.42, *p* = .016, *d* = 0.21. In contrast, participants in the control condition judged the young (*M* = 4.00) and older (*M* = 4.07) applicants similarly, *t*(1582)  = 0.85, *p* = .400, *d* = **−**0.07. This replicates the effect demonstrated by Uhlmann and Cohen (2007) in *both* the “objective” and “biased” priming conditions. Hiring discrimination increased both after agreeing that one is objective and failing to agree that one could be biased.

**Figure 1:**
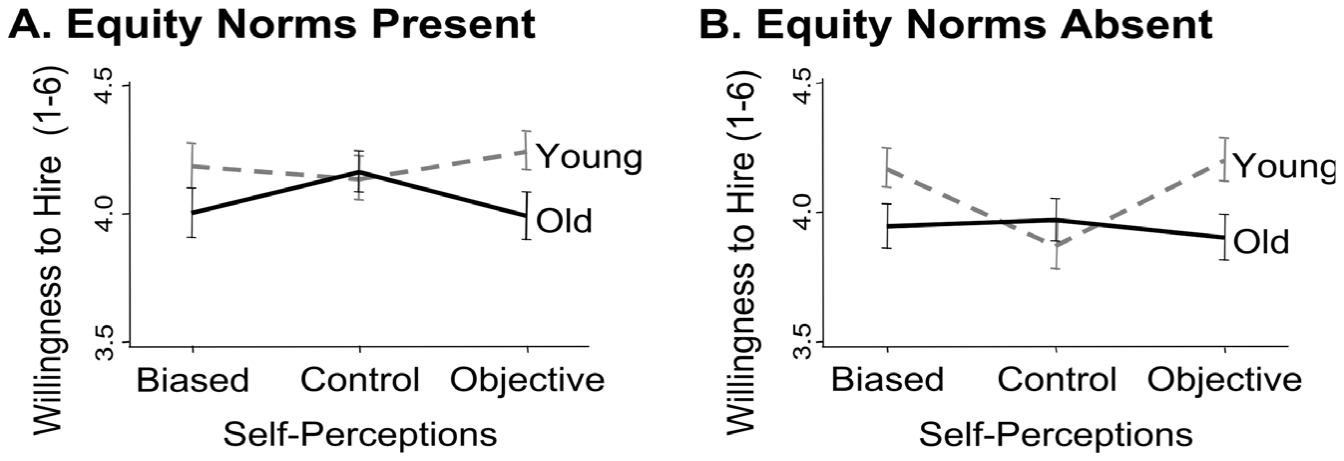
Mean differences in hiring decisions are represented as a function of whether the job applicant was young or old and of the self-perceptions experimental manipulation (biased, objective, or control), when equity norms were present (Panel A) or absent (Panel B). Error bars represent the standard errors.

**Presence of equity norms.** This pattern of effects held whether or not equity norms were present stating that hiring managers must comply with nondiscrimination statutes. However, the presence of equity norms *did* alter hiring decisions. As shown in Figure 1, subtly reminding participants of equity norms increased positivity for *all* job applicants, *F*(1, 1581)  = 5.11, *p* = .024, η_p_^2^ = .003, but did not interact with the applicants’ age, *F*(1, 1580)  = 0.01, *p* = .939; no other significant interactions qualified these results (all *F*s ≤ 1.50). That is, regardless of the applicants’ age, participants reported greater willingness to hire an applicant when equity norms were present (*M* = 4.13) than when equity norms were absent (*M* = 4.01). In short, the equity norms may have affected individuals’ motivation to respond favorably without making their decisions more *equitable*, as reflected by the persistent age gap in hiring preferences.

### Perceived Importance of Hiring Factors

We also evaluated whether either the presence of equity norms or self-perceptions priming affected participants’ post-decision evaluations of which criteria were used in their hiring decisions. The applicant materials held constant the applicants’ work experience, educational background, and marital and parental status. Additionally, the four applicant descriptions, which were randomized between participants, manipulated the applicants’ strength on creativity, interpersonal skills, dependability and productivity, and leadership skills. Thus, the decision factors that would be most likely to vary based on the experimental factors would be the relative importance of the applicants’ age and expertise to the hiring decision.

Despite the persistent age gap in hiring decisions, participants reported that the applicants’ age was only slightly important (*M* = 2.9), while the applicants’ expertise (*M* = 5.7) was very important, *paired t*(1520)  = 56.29, *p<*.001, *d* = 1.44. As hypothesized, the presence of equity norms increased the post-decision perceived importance of expertise (*d* = 0.37), a normative factor, and decreased the perceived importance of age (*d* = −0.12), a non-normative bias. These results suggest that equity norms increased individuals’ self-perceptions of what had influenced their behavior, without changing the actual discriminatory outcome.

**Applicants’ age.** The perceived use of the applicants’ age (R^2^  = .025) was simultaneously altered by significant main effects of all three manipulations, and no significant interactions qualified these results, all *F*s ≤ 3.11. That is, the applicants’ age was perceived as *more* important for older rather than young applicants, *F*(1, 1521)  = 26.22, *p<*.001, η_p_^2^ = .017, *d* = 0.27, *less* important when equity norms were present, *F*(1, 1521)  = 4.24, *p* = .040, η_p_^2^ = .003, *d* = **−**0.12, rather than absent, and differed significantly depending on the self-perceptions condition, *F*(2, 1521)  = 3.47, *p* = .031, η_p_^2^ = .005. Follow-up analysis with contrasts indicates that relative to the control condition, the applicants’ age was perceived as significantly less important in the self-perceived bias condition, *t*(1521)  = 2.51, *p* = .012, *d* = **−**0.16, and marginally less important in the self-perceived objectivity condition, *t*(1521)  = 1.95, *p* = .052, *d* = -0.12.

**Figure 2:**
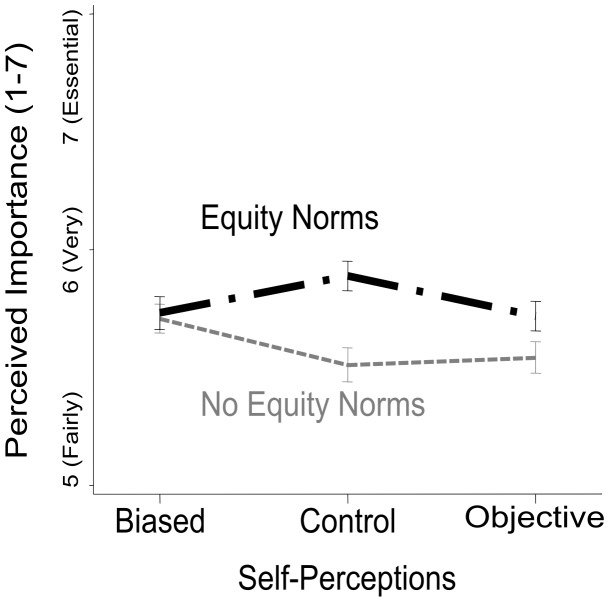
Mean differences in the perceived importance of the job applicants’ expertise in the hiring decision are represented as a function of the self-perceptions experimental manipulation (as either biased, objective, or control) and whether equity norms were present or absent; error bars represent the standard errors.

**Expertise.** The perceived importance of the applicants’ expertise (R^2^ = .016) was altered as a main effect of the presence of equity norms, *F*(1, 1514)  = 12.33, *p<*.001, η_p_^2^ = .008, and an interaction between equity norms and self-perceptions priming, *F*(2, 1514)  = 3.79, *p*  = .023, η_p_^2^ = .005 (see Figure 2) but was not altered by main effects of the applicants’ age, *F*(1, 1514)  = 2.26, *p* = .133, η_p_^2^ = .002, or self-perceptions priming, *F*(2, 1514)  = 1.06, *p* = .347, η_p_^2^ = .001; no interactions qualified these effects, all *F*s ≤ 1. Follow-up analysis of the simple main effect of equity norms in the priming conditions indicates that the presence of equity norms significantly increased the perceived importance of expertise in the control condition, *F*(1, 1514)  = 16.44, *p<*.001, *d* = 0.36, but did not significantly alter the importance of expertise in either the self-perceived objectivity condition, *F*(1, 1514)  = 3.32, *p*  = .069, *d* = 0.16, or the self-perceived bias condition, *F*(1, 1514)  = 0.04, *p*  = .8475, *d* = 0.02.

## Discussion

### Both Personal Objectivity and Equity Norms Influenced Hiring Decisions

In this volunteer sample of adults, individuals who were primed with a sense of their own objectivity preferred young rather than older job applicants with identical qualifications. In contrast, no age discrimination occurred among individuals in the control conditions. Notably, this effect was not qualified by the age of the participant – older participants were as likely to discriminate when primed with their own objectivity as were younger participants.

Given that a previous community-based audit study [3] found that older adults experienced substantially lower call-back rates than young applicants, the salient point is that self-perceived objectivity *increased* age discrimination, even in a tightly-controlled experimental setting. And while the presence of nondiscrimination statutes increased enthusiasm for all applicants *regardless* of their age, potentially by increasing *motivation* to respond favorably, it did not reduce age discrimination in hiring decisions. That is, reminding decision-makers of legal statutes barring discrimination failed to achieve those statutes’ stated purpose – to ensure nondiscrimination on the basis of age. The results also reflect the challenge of inducing a sense of personal *non*-objectivity. Individuals affirmed their own objectivity, but resisted acknowledging that their decisions could be less than fully objective.

Research on the bias blindspot emphasizes the strength of this belief, but suggests an alternative strategy for inducing a sense of personal nonobjectivity. For example, reading a supposed *Science* article describing the breadth of psychological research on how nonconscious processes influence judgments eliminated the bias blind spot – individuals’ tendency to perceive themselves to be much less susceptible than other people to various cognitive biases [21]. This would suggest that educating individuals about the power of nonconscious influences and the lack of introspective awareness to these biases might successfully challenge individuals’ sense of their own objectivity. But even if this strategy were successful, individuals might still express age discrimination. This possibility is suggested by theory [17] arguing that correcting for unwanted biases requires that the following series of conditions be fully met: individuals must be aware that the bias could influence their decision, be motivated to correct the bias, know the degree to which the bias influences their decision, and have the mental control necessary to adjust their decision. This highlights the difficulty of inducing individuals to recognize that they are subject to biases and to recognize biased judgments when they occur.

### Equity Norms Increased Casuistry in Perceived Importance of Hiring Criteria

Although individuals who primed with a sense of personal objectivity or bias demonstrated age discrimination, they believed that their hiring decisions were merit-based. Overall, individuals asserted that the applicants’ expertise was very important to their decision, while the applicants’ age was only slightly important. This fits with research on *casuistry,* which finds that when individuals demonstrate bias by selecting dominant-group members (e.g., in simulated hiring decisions or college admissions decisions), those individuals alter the perceived importance of selection criteria to justify their biased decision [19], [20].

The presence of an equal-opportunity statement prohibiting discrimination also further increased casuistry. When thinking about legal proscriptions on hiring based on age and other social group memberships, individuals reported their hiring decisions relied *more* on the applicants’ expertise and *less* on age. This is further evidence that equity norms increased *motivation* to respond without bias, but that individuals were unable to adequately identify and correct for age biases [17]. In further support for this idea, individuals saw themselves as being slightly *less* biased if they responded to the self-perceived bias questionnaire *after* the hiring decision rather than *before.* It also appears that when individuals were primed with a sense of their own objectivity, they found it less necessary to justify their decisions by reporting that the applicants’ expertise has been more influential to their decision.

Note that our equity norms manipulation specifically addressed the legal proscriptions of discriminating based on age or other social group memberships. An anonymous reviewer pointed out that other forms of communicating equity norms may not have similar effects as we observed here. These could be constrained to specific concerns about legal norms and responsibilities. When norms are communicated as community standards or popular values, it is possible that the effects could be different.

### Implications for Understanding Age Discrimination

The results from the present research highlight the power of individuals’ unawareness of their own biases and the difficulty that decision-makers experience in implementing equal-opportunity legislation. Discrimination can result from subtle indicators of applicants’ group memberships, such as the racial connotations of one’s first name [22] or parental status implied by volunteer activities [23] – indicators that decision-makers may not recognize as influencing their judgments. And even if decision-makers did recognize these biasing influences, they may not know how to correct for the biases [17]. The present results suggest that interventions that rely on decision-makers’ intentions to behave equitably may be ineffective. An alternative would be to alter the decision-making process itself to remove cues for irrelevant group memberships. For instance, when applicants auditioned for orchestra positions anonymously behind a screen, women’s chances of being hired increased dramatically [24]. In any case, simple changes to the mental context of decision-making – a mindset that one is objective or a reminder of nondiscrimination statutes – can influence both one’s hiring decision and one’s beliefs about how the decision was made. The challenge presented in these data is that these mental contexts increased or did not change discriminatory practices, but decision-makers appeared to think that they did.

## Supporting Information

Materials S1 **Materials for the hiring scenario.**
(DOC)
