# Supplementary material for: Age-Based Hiring Discrimination as a Function of Equity Norms and Self-Perceived Objectivity
Source: PLoS One. 2014 Jan 21;9(1):e84752. doi: 10.1371/journal.pone.0084752 (PMC3897397; doi:10.1371/journal.pone.0084752)
Supplement: Materials S1 — Materials for the hiring scenario. (DOC) [file pone.0084752.s001.doc]

Materials S1 – Hiring Decision Materials

*Imagine that you are part of a hiring committee for the following company. You will read about the traits and credentials of a job applicant and decide whether to hire the applicant. These traits may or may not be relevant to the decision of whether or not to hire the applicant. You may not always feel you have enough information to make a decision, but please do the best you can with the information provided.*

*[Equity norms, manipulating presence or absence] Keep in mind that hiring managers must follow state and federal laws prohibiting discrimination on the basis of age, disability, national or ethnic origin, race, religion, or sex.*

**Description of company & job:**

**Company X** is a high-end cosmetics and clothing manufacturer that is currently looking for a new Marketing Manager. This company produces hip, innovative products that define the latest styles and trends.

A **Marketing Manager** is responsible for directing and inspiring employees in the choice and design of marketing campaigns; for identifying potential customers and targeting advertising toward them; for monitoring trends in up-and-coming products; and for maximizing the company's profits while ensuring that customers are satisfied. The success or failure of the new manager will heavily influence company profits.

Now, carefully read this summary of a complete job application and evaluate the applicant's suitability:

| **INFORMATION** | **JOB APPLICANT** |
| --- | --- |
| **Name:** | Michael Jones |
| **Education:** | B.A., Purdue University, [Age manipulation: 1975 / 1999 ] |
| **Personal Information:** | - [Age manipulation: 54 / 31] years old - Married with one child |
| **Recent Experience:** | Marketing department in high-end cosmetics Company Y, with:   - 3 years experience: Marketing Specialist - 6 months experience: Interim Manager |
| **Highlights:  Recommendation #1** | - **[2 positive and 2 somewhat negative phrases, see Table 1 below]** |
| **Highlights:  Recommendation #2** | - **[2 positive and 1 somewhat negative phrases, see Table 1 below]** |

**Table 1**. Content of recommendation letter highlights for 4 counterbalanced job applicants

| **C#** | **Highlights:**  **Recommendation Letter 1** | **Highlights:**  **Recommendation Letter 2** |
| --- | --- | --- |
| #1 | - Was promoted to a position utilizing his interpersonal skills - Sometimes has difficulty making decisions - Voluntarily works overtime and takes work home to meet deadlines - Adequate performance in increasing departmental profits | - Interacts and gets along well with subordinates, superiors, and clients - Punctual and typically exceeds expectations - Occasionally hesitant in making a final decision regarding various subordinates’ ideas |
| #2 | - Has the analytical skills to identify needs and devise viable solutions - Required occasional guidance and mentorship in maintaining good relations with subordinates - Praised for being unflustered and productive during frenzied periods - Acceptable leadership skills | - Praised for how he handles pressure - Excellent capacity to quickly grasp new theories - Still improving grasp of interpersonal skills required for management |
| #3 | - Somewhat conservative in promoting/approving edgy marketing appeals - Remains steadfast in his cheerfulness, calmness, and dependability - Interacts and gets along well with fellow employees and superiors, as well as clients - Adequate, but not exceptional productivity | - Extremely positive and dependable employee - Exceeds expectations in interpersonal skills and dependability, but less so in creativity and vision - Attentive to tasks and works tirelessly to achieve the goals of the department |
| #4 | - Always willing to offer assistance and has an excellent rapport with employees and clients - Occasionally late for work and meetings - Excellent capacity to quickly grasp new theories and creatively generate related ideas - Disorganization sometimes hinders productivity | - Enjoys good relationships with employees and encourages their creativity - Productivity is occasionally hampered by lateness - Praised for innovative ideas in previous projects |

*Note.* The job applicant was described with one of four profiles of recommender highlights. These profiles were randomly-assigned across participants. Hiring evaluations were altered as a main effect of which profile was used to describe the job applicant, *F*(3, 1584) = 6.79, *p* = .0001, *R*^2^ = .013. But critically, the profile type never significantly interacted with the manipulated factors, as self-perceptions manipulations, the presence of equity norms, and the applicants’ age (all *F*s ≤ 2.23). Nor did it interact significantly with the manipulated factors in predicting the perceived importance of the applicants’ age (all *F*s ≤ 1.49) or the perceived importance of the applicants’ expertise (all *F*s ≤ 1.85).
